# Antibacterial Effect of *Luma apiculata* (DC.) Burret Extracts in Clinically Important Bacteria

**DOI:** 10.1155/2019/7803726

**Published:** 2019-10-13

**Authors:** Tiare Araya-Contreras, Rhonda Veas, Carlos A. Escobar, Pamela Machuca, Mauricio Bittner

**Affiliations:** ^1^Laboratorio de Microbiología y Biotecnología Oral, Departamento de Ciencias de la Vida, Facultad de Ciencias de la Vida, Universidad Andres Bello, Santiago, Postal Code 8370133, Chile; ^2^Laboratorio de Síntesis Orgánica y Organometálica, Departamento de Ciencias Químicas, Facultad de Ciencias Exactas, Universidad Andres Bello, Av. República 330, Santiago, Postal Code 8370133, Chile; ^3^Facultad de Odontología, Universidad Andres Bello, Santiago, Postal Code 8370133, Chile

## Abstract

Nosocomial infections caused by bacteria are one of the main public health problems. Moreover, the resistance to antibiotics by these bacteria makes it necessary to find new treatments to fight them. *Objective*. To evaluate the antibacterial activity of *Luma apiculata* (DC.) Burret extracts on bacteria of clinical importance. *Materials and Methods*. In this study, extracts were obtained at room temperature by successive extraction of *L. apiculata* leaves, flowers, and branches and treated separately with solvents of ascending polarity (i.e., hexane, methylene dichloride, ethyl acetate, ethanol, methanol, and water) to extract the compounds depending on their polarity. Then, the extract's antibacterial activity was tested against *Staphylococcus aureus*, *Staphylococcus epidermidis*, *Staphylococcus saprophyticus*, *Enterococcus* sp, *Acinetobacter baumanii*, *Pseudomonas aeruginosa*, and *Escherichia coli*. *Results*. The hexane extract of *L. apiculata* leaves resulted to be active against all bacteria tested. Among them, *S. aureus* showed to be the more susceptible, showing a minimum inhibitory concentration (MIC) of 120 *μ*g/ml. In addition, a growth curve was performed, and colonies were counted. A decrease in bacterial growth was observed when the hexane extract of *L. apiculata* leaves was added. Besides, the hexane extracts of *L. apiculata* flowers resulted to be active against all Gram-positive tested bacteria. However, at higher concentrations, this extract resulted inactive for the Gram-negative bacteria tested. The hexane extract of *L. apiculata* branches resulted to be inactive in all cases. The extracts obtained treating separately leaves, flowers, or branches with solvents of major polarity than the hexane in a successive extraction of ascending polarity methodology resulted also to be inactive as an antimicrobial against all bacteria tested. *Discussion/Conclusion*. The hexane extract of *L. apiculata* leaves showed the lower MIC against *S. aureus* when compared with extracts obtained from other parts of the plant. The growth curve and the colonies count suggest a bacteriostatic activity of the *L. apiculata* leaves extract against *Staphylococcus aureus*.

## 1. Introduction

Through time, humans have tried to relieve their ailments and prolong life. Even though humans in the XXI century are still trying, nobody has been able to avoid death, limiting only to mitigate symptoms of diseases and avoiding the development of others [1]. The environment offers an extended variety of medicinal plants, which are used since ancient times to improve people's health and cure all kinds of diseases. In fact, it is believed that up to 25,000 medicinal plants are used with therapeutic purposes worldwide. Phytotherapy is defined as the use of medicinal plants and their derivatives for therapeutic purposes in order to prevent, relieve, or cure diseases [2]. In Chile, the use of medicinal plants dates from remote times, where native Chilean people, such as the Mapuche, inherited most of the current knowledge in ethnobotany. There are several plants that can be mentioned as an example of plant's use in natural medicine by natives. For instance, chamomile (*Matricaria recutita*) infusions made by flowers and leaves are still used as a digestive treatment, matico (*Buddleja globosa*) is used as a wound healer, or Maitén (*Maytenus boaria*) as an antipyretic [3, 4]. In addition, in the province of Yucatan, Mexico, the use of medicinal plants dates to Mayan culture, where natives commonly use plant species such as *Aloe vera*, *Ocimum campechianum*, *Tithonia diversifolia*, *Ruta chalepensis, Tradescantia spathacea, Parthenium hysterophorus, Chromolaena odorata*, *and Cissampelos pareira*. [5].

*Luma apiculata* (DC.) Burret is used by Mapuche people as an antiasthmatic, antidiarrheal, and antiseptic. Interestingly, the cortex of this plant is boiled and used as an antiseptic in washes described to treat wounds and ulcerations. This tree is a native species of Chile (although it might also be found in Argentina). It belongs to the Myrtaceae family, and it is commonly known as Arrayán. It spreads among the central and south regions of Chile, extending from Coquimbo to Aysén [6], can reach up to 15 meters high, and it is characterized by its red-colored bark, its white flowers, and black fruits that can be observed during summer and autumn.

One of the most relevant pathogenic bacteria is those related to intrahospital or nosocomial infections. These microorganisms are defined by the World Health Organization (WHO) as infections that occur during or as a result of hospitalization, not present or incubating at the time of patient admission. The main bacteria associated with these infections are: *Pseudomonas* spp, a Gram-negative nonfermenting bacillus related to urinary tract infections (UTI) in which mortality ranges from 35% to 70% depending on the location of the infection [7]; *Escherichia coli*, a Gram-negative bacillus which belongs to the *Enterobacteriaceae* family, also related to urinary tract infections (UTI) [8]; *Acinetobacter baumannii* a Gram-negative nonfermenting coccobacillus related to infections of the bloodstream and pneumonias associated with mechanical ventilators and represents 80% of infections [9, 10]; *Enterococcus* spp. a Gram-positive coccus frequently found in urinary tract infections and wound infections after surgeries [11] and; *Staphylococcus aureus*, a Gram-positive facultative anaerobe round-shaped bacterium generally grouped in clusters of irregular shape—also capnophilic, nonmotile, with a polysaccharide capsule that helps bacterium to avoid phagocytosis and facilitates their capacity to resist adverse conditions such as heat or high salinity (7.5% of NaCl) [12]. *S. aureus* is considered the most dangerous pathogen not only in nosocomial infections in humans but also due to many other diseases. It produces superficial lesions on the skin and abscesses and other kinds of injuries including osteomyelitis, invasive endocarditis, septic arthritis, and septicemia [13]. Considering the aforementioned information, the purpose of this work was to evaluate the antimicrobial effect of different extracts of *L. apiculata* on bacteria of clinical importance, especially over *Staphylococcus aureus*.

## 2. Materials and Methods

### 2.1. Collecting, Processing, and Obtaining Extract of Biological Material

The plant material was collected at SENDA Darwin biological station located in the large island of Chiloe, X region, Chile (42°53 ′S, 73°39′ W). The exhibition deposit was made at the National Museum of Natural History (MNHN) Code: SGO 165692 and SGO 165693. It was collected in February, taking care to separately collect leaves, flowers, and branches.

Extracts of L. *apiculata* leaves, flowers, and branches were obtained at room temperature soaking separately dried leaves, flowers, or branches successively in solvents of ascending polarity: hexane, methylene dichloride, ethyl acetate, methanol, ethanol, and water as will be described in the following paragraph [14].

The plant materials were dried at room temperature under controlled ventilation. Once dry, the plant materials were ground with a food processor. The dried ground plant materials (i.e., leaves, flowers, or branches treated separately) were weighed and placed in an Erlenmeyer flask, covered with hexane (ratio 1 : 10 w/v) and allowed to extract for 48 h at room temperature. Then, the solvent containing the extract was filtered with a Whatman No. 2 filter paper, and the liquid fraction was collected and concentrated *in vacuo* to obtain the hexane extract. The hexane extract was dried in the vacuum pump to eliminate the residual solvent, and the remaining plant material was submitted to a new extraction. To do that, residual hexane remaining in the extracted ground plant materials was dried at room temperature under an extraction hood. Once dry, the same procedure described above was repeated but this time using methylene dichloride. Equally, the procedure was repeated using successively ethyl acetate, methanol, ethanol, and finally, water.

To calculate the extract yield, the following formula was used:

(1) $$\begin{array}{c} \frac{\text{extract dry weight}}{\text{plant material weight}}\;\times 100. \end{array}$$

Subsequently, the dried extracts, obtained either in powder or paste, were redissolved in their extraction solvent (to ensure solubility) generating, in this way, a set of six stock solutions at a concentration of 10 mg/mL (one stock for each extracting solvent used). From each stock, new diluted solutions were prepared in the corresponding extraction solvent to obtain concentrations ranging from 1 mg/mL to 1 *μ*g/mL. The stock solution, as well as its dilutions, was stored at 4°C until its use. Bars depict the average values from three independent experiments.

### 2.2. Determination of Minimum Inhibitory Concentration

The bacterium was incubated in a plaque for 24 h at 37°C. At the end of this period, isolated colonies were taken and resuspended in sterile physiological saline solution to obtain a Mc Farland turbidity grade 0.5. In glass tubes, liquid culture medium (MH ideally) was added, and the bacterial suspension was inoculated in a 1 : 1000. In each tube, an extract was added at concentrations of 10 mg/mL, 8 mg/mL, 6 mg/mL, 4 mg/mL, 2 mg/mL, and 0.2 mg/mL, to obtain final concentrations of 500, 400, 300, 200, 100, and 10 *μ*g/mL, respectively. As control was used: control broth, broth with bacteria, and broth with the extract. It was incubated 24 h at 37°C, to observe the presence or absence of turbidity. The hexane solvent was used as a negative control. Bars depict the average values from three independent experiments [15].

### 2.3. Disk Diffusion Test

Gram-positive bacteria (*Staphylococcus aureus, Staphylococcus epidermidis, Staphylococcus saprophyticus,* and *Enterococcus* sp), as well as Gram-negative (*Acinetobacter baumanii*, *Pseudomonas aeruginosa*, and *Escherichia coli*), were incubated on BHI plaques for 24 h at 37°C. At the end of this time, isolated colonies were taken and resuspended in sterile physiological saline solution to obtain an Mc Farland turbidity grade 0.5. With the suspension, a bacterial lawn was performed in Müller Hinton medium with a sterile swab and cotton disks previously loaded with different concentrations of extract mixed with 2 *μ*L DMSO were placed. Finally, they were incubated for 24 h at 37°C and, then, the appearance of an inhibition halo was observed. The hexane solvent was used as a negative control. Bars depict the average values from three independent experiments [16].

### 2.4. Growth Curve Test and Counting Colony-Forming Units (CFU/mL)

In a tube with BHI broth, a suspension of *S. aureus* was performed with an Mc Farland 1, whereby a new culture was inoculated at a ratio of 1 : 250, which was used to perform the assay. The optical density was measured at 600 nm every 1 hour until the exponential phase was reached, and then, it was measured every half hour. Colony-forming unit per mL was determined as follows: Counts were performed each hour and then, when the exponential phase was reached, counts were every half hour. The bacterium was used as a positive control without adding extract. Bars depict the average values from three independent experiments:

(2) $$\begin{array}{c} \text{counting colony}-\text{forming units}\left(\text{CFU}/\text{mL}\right)=\text{number of colonies }*\text{ dilution factor }*\;200. \end{array}$$

### 2.5. Sensitivity Test of *Luma apiculata* Extracts in Clinical Strains of *Staphylococcus aureus* Isolated from Patients Suffering Palatine Cleft Lip

Samples were isolated from voluntary patients suffering palatine cleft lip because in them there is an increased incidence of these intraoral microorganisms due to direct propagation through the fissure. Samples were plated on blood agar medium and after incubation for 24 h at 37°C. To isolate the *Staphylococcus* strains from the patient's bacterial flora, the blood agar obtained colonies were isolated, chopping the blood agar containing the *Staphylococcus* colony and placing it in the selective salty mannitol culture medium. The strains grown in this medium (positive for mannitol) were confirmed through macroscopic analysis of colony morphology, through microscopic analysis by Gram stain and biochemical tests (catalase test, SLIDEX Staph plus). The sensitivity test for *S. aureus* isolated from clinical patients suffering palatine cleft lip was also performed as follows: antimicrobial Müller–Hinton medium with 5 *μ*L extract of *L. apiculata* 1 mg/mL, using antibiotic penicillin as control, and 5 *μ*L of hexane (extract solvent). The assay was incubated at 37°C for 24 hours.

### 2.6. Statistical Analysis

All statistical analyses were performed with the SPSS Statistics V23.0 program and Minitab V10.0.

## 3. Results and Discussion

During the past decades, methicillin-resistant *S. aureus* has been the leading cause of a wide range of clinical infections [17], which includes infections such as cellulitis, pyomyositis, arthritis, and osteomyelitis [18]. *S. aureus* has shown resistance to antibiotics, such as phenoxymethylpenicillin (87.1%), azithromycin (11.6%), and erythromycin (11.2%) [19], being vancomycin currently the main drug used to treat most diseases associated with methicillin-resistant *S. aureus* [20]. In this context, the search for new leader structures originated from plants to obtain new and more powerful compounds that allow the control of resistant infectious organisms is becoming increasingly necessary. This search of novel chemical structures has led scientist to intensify the exploration for secondary metabolites present in plants, including all types of plant organs (leaves, stems, roots, flowers, and fruits) [21]. In this work, a total of 18 extracts were obtained at room temperature by successive extractions of *L. apiculata* leaves, flowers, and branches, treated separately with solvents of ascending polarity (i.e., hexane, methylene dichloride, ethyl acetate, methanol, ethanol, and water). In addition, the yield of the leaf's extracts was calculated with all the solvents described above (Tables 1 and 2). Their antimicrobial activity was tested against *Staphylococcus aureus, Staphylococcus epidermidis, Staphylococcus saprophyticus, Enterococcus* sp*, Acinetobacter baumanii, Pseudomonas aeruginosa*, and *Escherichia coli*. The hexane extract of *L. apiculata* leaves resulted to be active against all bacteria tested. Among them, *S. aureus* showed to be the most susceptible to leaves-hexane extract. To be sure that the antimicrobial activity obtained was coming from the extract and not from the solvent, a test on Müller–Hinton agar was carried out to rule out the antimicrobial action of the pure hexane. These assays revealed that hexane showed no antimicrobial activity (data not shown) against all bacteria tested [22].

In addition, the hexane extracts of *L. apiculata* flowers resulted to be active against all Gram-positive bacteria tested but only at higher concentrations. However, this extract resulted to be inactive for all Gram-negative bacteria tested, as well as the hexane extract from *L. apiculata* branches. Since flowers appear in nature only between December to March [23] and the extract resulted partially active (very high concentration to show antimicrobial activity), we discard their use as an antimicrobial source. Furthermore, we discarded the extracts from leaves, flowers or branches obtained with solvents of major polarity than hexane, because these extracts had no antimicrobial effect against all bacteria tested.

**Figure 1:**
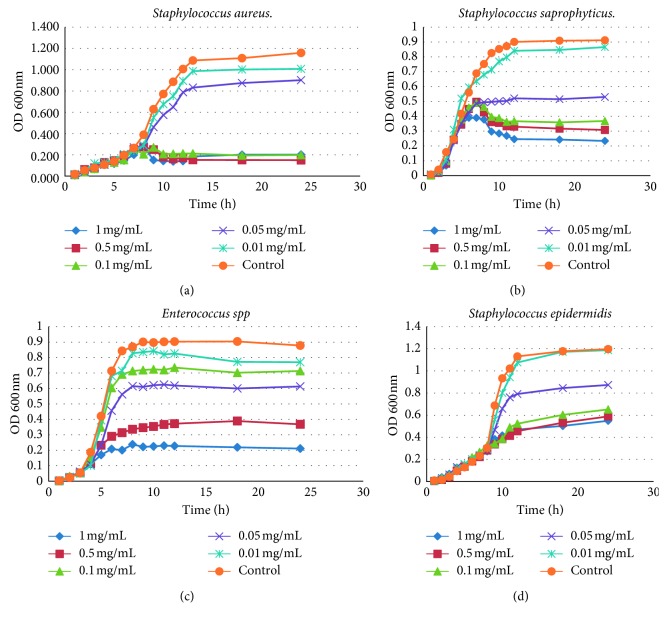
Antimicrobial activity in BHI medium leaf extract *Luma apiculata* on growth curves of Gram-positive bacteria concentrations: 1 mg/ml, 0.5 mg/ml, 0.1 mg/ml, 0.05 mg/ml, and 0.01 mg/ml (*n*=3).

Since only the hexane extract from *L. apiculata* leaves showed a promissory antibacterial activity at low concentrations against *S. aureus*, *S. saprophyticus*, *S. epidermidis*, and *Enterococcus* spp, growth curves were performed confronting bacteria with dilutions of the extract. As a result, we found that the growth curve of *Luma apiculata*'s leaves-hexane extract has a range of effect between 0.1 mg/mL and 1 mg/mL (Figure 1) against all Gram-positive bacteria used. Notwithstanding, it has a greater effect against *S. aureus*, since it has a considerable decrease in bacterial growth with a concentration of 0.1 mg/mL.

Once the antimicrobial activity on *Staphylococcus aureus* was observed, the minimum inhibitory concentration in liquid medium was determined for the leaves-hexane extract. It was established that the minimum inhibitory concentration was between 100 *μ*g/mL and 150 *μ*g/mL; the intermediate concentrations were subsequently evaluated, and it was established that the minimum inhibitory concentration was found at 120 *μ*g/mL. As a control, the same general procedure was followed to determine the minimum inhibitory concentration for the flowers-hexane extract against *S. aureus*, being 300–350 *μ*g/mL, the minimum concentration found in this case, showing, as observed before, that flowers-hexane extract is less effective, since a higher concentration is required to obtain the same effect against *S. aureus*. The same procedure was used to estimate the minimum inhibitory concentration of the flower-hexane and leaves-hexane extracts, and the results are summarized in Table 2. The results of the present study can be compared with the data obtained by Tekwu et al. [24], who evaluated antimicrobial activity against *S. aureus* from hexane extracts of *Albizia gummifera*, obtaining a minimum inhibitory concentration of 512 *μ*g/mL and 256 *μ*g/mL for extracts from *Ficus exasperata*. When comparing the minimum inhibitory concentrations, it is possible to evidence that *Luma apiculata* has a lower antimicrobial concentration, thus, it is more active than the other plant species previously described. In addition, compared to the extract of *Syzygium antisepticum*, a plant of the same family as *L. apiculata*, it was observed that both are effective against *S. aureus* with a similar minimal inhibitory concentration (between 120 and 130 g/mL) [25].

Table 3 summarizes the results of antimicrobial effect of the hexane extract against *S. aureus* strains obtained from patients suffering palatine cleft lip. Moreover, Table 3 summarizes some enzymatic characteristics of *S. aureus*, as well as the strains' susceptibility to penicillin. As observed, all tested strains were susceptible to *Luma apiculata*'s hexane extract regardless of antibiotic resistance.

**Figure 2:**
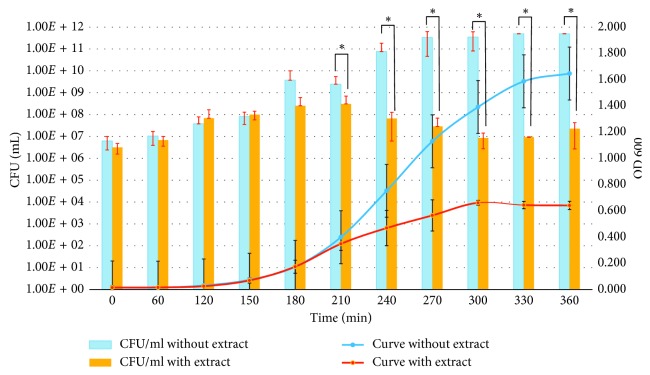
Growth curve and count of colony-forming units of clinical strains of *Staphylococcus aureus*. The light blue bars correspond to the colony count without the extract of *Luma apiculata* 1 mg/mL. The yellow bars correspond to the colonies count with *Luma apiculata* extract of 1 mg/mL. In blue, the curve without extract of *Luma apiculata* is observed. In red, the curve is observed with extract of *Luma apiculata* of 1 mg/mL. ^*∗*^The extract of *Luma apiculata* was incorporated after 150 minutes; *n*=3 (^*∗∗*^*p* < 0.005; ^*∗*^*p* < 0.05).

In order to establish whether antimicrobial effect was bacteriostatic, bactericidal or bacteriolytic, a growth curve was performed and bacterial counting in presence and absence of the extract [26]. *L. apiculata*'s hexane extract was added at a concentration of 1 mg/mL when bacteria reached early exponential growth phase.  Figure 2 shows a bacteriostatic behavior since turbidity and counts were maintained through time compared to the negative control (i.e., curve without added extract [27]).

Since the tree maintains its forage throughout the year as it is a perennial tree with a high frequency of leaf renewal [28], the use of leaves to look for antimicrobial activity seems to be an advantage since it would allow the use of the leaves in therapy without damaging the tree or the habitat.

## 4. Conclusion

Hexane leave's extract of *L. apiculata* showed an antibacterial effect on both Gram-positive and Gram-negative bacteria. In addition, the same extract resulted to be more effective against *Staphylococcus aureus* even when compared with other species of the same bacteria genus. For *S. aureus*, a minimum inhibitory concentration (MIC) of 120 *μ*g/mL was established.

When performing the growth curve and the count of colony-forming units (CFU/mL) of *Staphylococcus aureus* in the presence of *Luma apiculata*'s hexane extract, an arrest of bacterial proliferation was observed, suggesting a bacteriostatic activity against *Staphylococcus aureus.*

The *L. apiculata*'s hexane extract of leaves has an antibacterial effect on bacteria Gram-negative and Gram-positive. In addition, the *L. apiculata*'s hexane extract of leaves was more effective for *Staphylococcus aureus*, even over species of the same genus of bacteria. This means, a minimum inhibitory concentration (MIC) was established at 120 *μ*g/mL.

When performing the growth curve and the count of colony-forming units (CFU/mL) of *Staphylococcus aureus* in the presence of *Luma apiculata*'s hexane extract, an arrest of bacterial proliferation was observed, suggesting a bacteriostatic activity against *Staphylococcus aureus.*

## Figures and Tables

**Table 1:** Yield and characteristics of leave's extracts with different solvents.

Solvents	Yields	Characteristics
Hexane	0.5%	Pasty texture, yellow, strong smell
Methylene dichloride	1.1%	Pasty texture, dark green, strong smell
Ethyl acetate	0.5%	Grainy texture, black, odorless
Ethanol	2.1%	Grainy texture, dark brown, strong smell
Methanol	1.9%	Caramelized texture, dark brown, odorless
Water	3.5%	Grainy texture, dark brown, soft smell

**Table 2:** Antimicrobial effect of different extracts of *Luma apiculata* in bacteria of clinical importance.

Solvent	Extract	Gram positive				Gram negative		
		*Staphylococcus aureus*	*Staphylococcus epidermidis*	*Staphylococcus saprophyticus*	*Enterococcus* sp	*Acinectobacter boumanii*	*Pseudomonas aeruginosa*	*Escherichia coli*
Hexane	Leaves	>100 *μ*g/ml	>200 *μ*g/ml	>200 *μ*g/ml	>300 *μ*g/ml	>400 *μ*g/ml	>500 *μ*g/ml	>400 *μ*g/ml
	Branches	N/E				N/E		
	Flowers	>300 *μ*g/ml	>500 *μ*g/ml	>500 *μ*g/ml	>300 *μ*g/ml	N/E		

Methylene dichloride	Leaves	N/E				N/E		
	Branches							
	Flowers							

Ethyl acetate	Leaves	N/E				N/E		
	Branches							
	Flowers							

Ethanol	Leaves	N/E				N/E		
	Branches							
	Flowers							

Methanol	Leaves	N/E				N/E		
	Branches							
	Flowers							


Water	Leaves	N/E				N/E		
	Branches							
	Flowers							

^*∗*^N/E: no effect.

**Table 3:** Characterization of clinical isolates of *Staphylococcus aureus* patient palatal lip.

Strain	Gram	Catalase	Mannitol	Sensitivity to extract of *Luma apiculata*	Sensitivity to penicillin (10 *μ*g)
**109**	+	+	+	S	R
**116**	+	+	+	S	I
**121**	+	+	+	S	R
**125**	+	+	+	S	R
**147**	+	+	+	S	R
**149**	+	+	+	S	R
**150**	+	+	+	S	R
**182**	+	+	+	S	R
**188**	+	+	+	S	R
**189**	+	+	+	S	R
**192**	+	+	+	S	R
**208**	+	+	+	S	R
**288**	+	+	+	S	S
**520**	+	+	+	S	R
**545**	+	+	+	S	R
**566**	+	+	+	S	S
**598**	+	+	+	S	R

^*∗*^Sensitivity: S (sensitive), R (resistant), and I (intermediate).

## Data Availability

The data used to support the findings of this study are included within the article, and any other data needed to support the funding of the study are available from the corresponding author upon request.
